# Attentional Bias Modification Training Based on Virtual Reality and Eye Tracking in Anorexia Nervosa Patients

**DOI:** 10.3390/jcm12185932

**Published:** 2023-09-12

**Authors:** Mariarca Ascione, Marta Carulla-Roig, Helena Miquel-Nabau, Bruno Porras-Garcia, Franck-Alexandre Meschberger-Annweiler, Eduardo Serrano-Troncoso, Marta Ferrer-Garcia, Manuel Moreno-Sánchez, Jose Gutierrez-Maldonado

**Affiliations:** 1 Department of Clinical Psychology and Psychobiology, Institute of Neurosciences, University of Barcelona, Passeig de la Vall d’Hebron 171, 08035 Barcelona, Spain; ascione.m@ub.edu (M.A.); helena.mn29@gmail.com (H.M.-N.); franck.meschberger@ub.edu (F.-A.M.-A.); martaferrerg@ub.edu (M.F.-G.); 2 Department of Child and Adolescent Psychiatry and Psychology, Hospital Sant Joan de Déu of Barcelona, Passeig de Sant Joan de Déu, 2, Esplugues de Llobregat, 08950 Barcelona, Spain; marta.carulla@sjd.es (M.C.-R.); eduardo.serrano@sjd.es (E.S.-T.); 3 Department of Population Health Science, University of Utah School of Medicine, 295 Chipeta Way, Salt Lake City, UT 84112, USA; brnopg91@gmail.com; 4 Department of Cognition, Development and Educational Psychology, University of Barcelona, Passeig de la Vall d’Hebron 171, 08035 Barcelona, Spain; manelsg@gmail.com

**Keywords:** anorexia nervosa, attentional bias modification, body dissatisfaction

## Abstract

Anorexia nervosa (AN) patients exhibit attentional bias (AB) related to the body, which is the tendency to pay greater attention to weight-related body areas compared to non-weight-related ones. This phenomenon has been linked to elevated levels of body dissatisfaction (BD) and may potentially reduce the effectiveness of body exposure therapy. The purpose of this pilot study is to assess the efficacy of a single session of a new body-related AB modification task (ABMT) that combines virtual reality with eye tracking in patients with AN. The goals of the ABMT are to reduce body-related AB by balancing attention between weight and non-weight-related body areas and to reduce BD levels. Twenty-three adolescent patients with AN were embodied in a virtual avatar and immersed in a virtual environment where they completed the ABMT. Body-related AB measures and BD levels were assessed before and after the training. A paired samples *t*-test showed statistically significant differences between pre-assessment and post-assessment; the complete fixation time on weight-related body parts was reduced and BD levels decreased. The initial evidence of the efficacy of this ABMT has important clinical implications, since AB and BD are considered risk factors for developing and maintaining eating disorder symptomatology among patients with AN.

## 1. Introduction

Anorexia nervosa (AN) is a serious eating disorder with high mortality rates [1] and is often diagnosed in early adolescence or adolescence [2]. Neurocognitive deficits including attentional bias (AB) are implicated in developing and maintaining eating disorders [3,4]. AB is a phenomenon that is defined as the tendency to focus attention to information perceived as threatening over other types of information in response to a stimulus related to the disease [5,6]. It has been found that most patients with AN show an AB toward their body, focusing more attention on disliked body parts or weight-related body parts (e.g., stomach, thighs) and ignoring body parts not related to weight (e.g., neck, arms) [7]. A cognitive approach to psychopathology identifies AB as the result of maladaptive cognitive processes and schemas related to appearance, shape, and weight [5,8]. The patient’s way of thinking and behaving is constantly determined by such schemas. They automatically process only body information that is consistent with their dysfunctional self-schema (related to “fat”) and ignore the schema with inconsistent information (related to “thin”) [5].

Body-related AB is an important causal and maintenance factor in body dissatisfaction (BD) [9,10,11] and is one of the prominent risk and maintenance factors for AN [12]. Indeed, numerous findings indicate that AB to body-related stimuli is moderated by the degree to which an individual self-reports BD [13]. Additionally, it has been found that dysfunctional body-related AB could interfere with and reduce the efficacy of exposure-based treatment, such as mirror exposure therapy, which is generally used in patients with AN to treat body image disturbances and improve the results of classic cognitive-behavioral therapy [14]. One study shows that patients who had higher AB toward weight-related body parts were those who benefited least from MET (Mirror exposure therapy) [15]. The objective of MET is to look at all parts of the body for the same amount of time. However, these patients may tend to look excessively at weight-related body areas and neglect other parts of the body, which makes MET less effective [15].

Reducing body-related AB is clinically important for preventing and treating eating disorders [16]. Cognitive theories suggest that incorporating AB modification training (ABMT) into the treatment of AN can improve attentional control [16,17]. Repeated practice of ABMT leads to neuroplasticity-mediated changes in the brain, modifying automatic cognitive processes [16]. ABMT has shown effectiveness in various psychological conditions, including anxiety disorders, depression, addictive disorders, obsessive compulsive disorders, and eating disorders [18]. While ABMT has been used to reduce AB toward food-related stimuli in eating disorders [19,20,21], no studies have explored its use with body-related stimuli. Currently, five studies have utilized traditional ABMT to address body image concerns in non-clinical samples [22,23,24,25,26]. The most utilized technique is the modified probe detection task, originally adapted from MacLeod et al. [27] and coupled with eye-tracking (ET) devices [26] in some instances. However, this traditional technique, often conducted on desktop computers or smartphones, may lack ecological validity [28]. The probe detection task is based on the repetitive presentation of single pairs of stimuli or relatively complex patterns of stimuli, like images of self-defined attractive and unattractive body parts [26] or words that concern appearance, body shape, and food [22,23,24,25] to divert attention away from disorder-related stimuli. The nature of these stimuli may not fully capture the complexity of real-life situations [28]. Additionally, the repetitive nature of these tasks may lead to decreased participant engagement and reduced attentional control, potentially diminishing their effectiveness in modifying biases or symptoms [29].

To overcome these limitations, one potential solution is to integrate virtual reality (VR) and eye-tracking (ET) technologies. VR is increasingly used in eating disorder treatment to improve dysfunctional eating behaviors and body image disturbances [30,31] by immersing patients in realistic simulations that replicate their bodies and real-life situations [32,33], evoking emotions and reactions similar to real-life experiences while providing a safe and controlled setting [30]. ET technology facilitates accurate and continuous measurement of eye positions and movement throughout tasks. This technology provides a high level of precision, allowing for detailed tracking and analysis of gaze patterns [34]. VR and ET are often used separately. However, integrating them provides a whole new way to interact with VR content and improve the overall virtual experience.

Given the documented effectiveness of VR and ET-based ABMT in reducing body-related AB in healthy women [35,36], it is reasonable to explore the potential benefits of applying this approach to adolescents with AN. Early treatment is crucial for adolescents with AN due to their increased risk of long-term health complications and higher suicide risk, as it leads to improved long-term outcomes and a higher likelihood of achieving full recovery [37]. This study proposes the hypothesis that implementing ABMT aimed at promoting balanced attention towards the entire body will effectively reduce body-related AB. It is anticipated that this reduction in AB will correspond to lower levels of BD.

## 2. Materials and Methods

### 2.1. Clinical Sample

This study included twenty-three female adolescent patients (age = 15.30 ± 1.29 years; BMI = 18.28 ± 1.62 kg/m^2^) from the eating disorders unit of Hospital Sant Joan de Déu of Barcelona. Inclusion criteria consisted of a primary diagnosis of AN according to the DSM-5 [38], an age between 12 and 17 years and 11 months, and classification as underweight based on body mass index (BMI) for age and sex growth references charts [39]. Exclusion criteria were severe mental disorders with manic or psychotic symptoms, epilepsy, sensory complications preventing exposure, pregnancy, and clinical cardiac arrhythmia. All of the patients received a multidisciplinary approach treatment, including biological management, nutritional rehabilitation, behavioral programs to improve eating habits and weight, cognitive therapy (individual and group), and counseling for both individuals and parents. The majority received an intensive day program for 11 h a day (sleeping at home), while only one was in outpatient care, which is suitable for patients with good compliance and no significant risk factors. Intensive day hospital care is usually reserved for cases where there is no improvement in weight or eating behavior, especially when physical health is severely compromised or comorbid psychopathology is present.

### 2.2. Measures

Body dissatisfaction. BD was assessed using the Body Image Assessment Scale-Body Dimensions (BIAS-BD) [40]. This figural drawing scale questionnaire consists of a series of 17 silhouettes that depict a range of body sizes, spanning from 60 to 140 percent of the average female BMI. The pre- and post-training assessments utilized two different test-retest versions (A and B), with randomized silhouettes to mitigate any potential order effect bias. Participants chose the silhouette that best represented their current body size and the one that reflected their ideal body size. BD was determined by calculating the difference between the perceived body size and the self-defined ideal body size. Scores close to 0 represent no desired body change, and larger scores represent larger desired body change. This scale exhibits good psychometric properties, demonstrating robust test-retest reliability (r = 0.86) and substantial concurrent validity (r = 0.76) [40].

**Figure 1:**
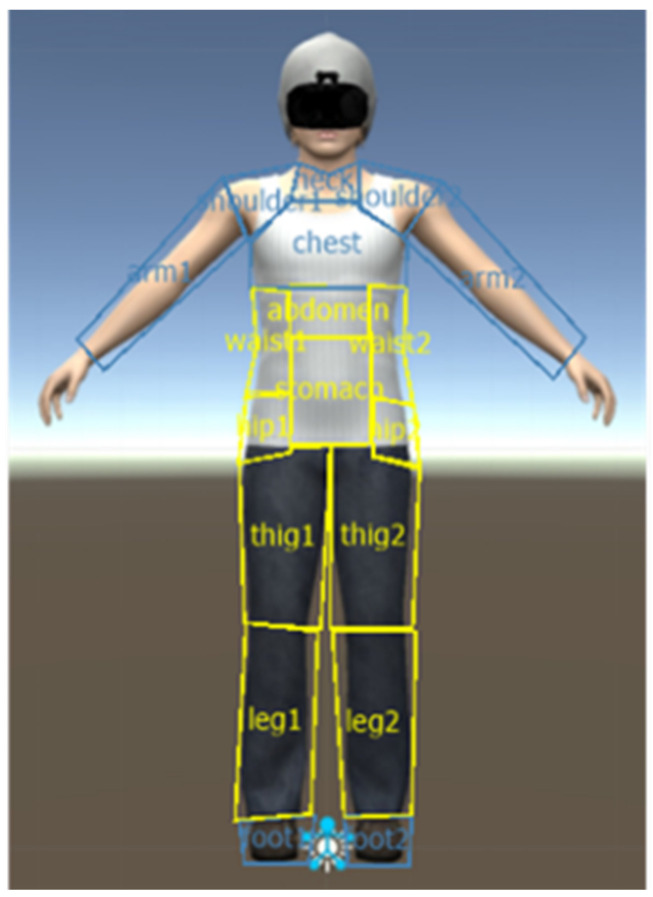
Weight-related areas of interest are delineated in yellow, while non-weight-related areas of interest are demarcated in blue on the female virtual avatar employed by OGAMA software (Version 5.1) to analyze attentional bias related to the body.

Body-related attentional bias measures. Visual fixation on the virtual body served as a measure of AB. The body was divided into two areas of interest (AOIs) based on a categorization derived from the Physical Appearance State and Trait Anxiety Scale [41] (see Figure 1):

• Weight-related AOIs encompassed body regions commonly associated with measures of eating disorders, including the stomach, hips, waist, thighs, and legs.
• Non-weight-related AOIs included body parts less correlated with eating disorders: neck, chest, shoulders, arms, and feet.

In this study, the participant’s head was not taken into account as the avatar’s head was covered by a head-mounted display, which was also worn by the participant. Visual fixation, defined as the behavior of sustaining one’s gaze on a specific location for a minimum duration, typically 100–200 ms [42], was assessed using two reliable and continuous measures [33,43,44]: the number of fixations, which represents the total count of fixations on the specified group of the AOI, and complete fixation time, which refers to the cumulative duration of fixations on the specified AOI group in milliseconds.

### 2.3. Instruments

Hardware. The VR system utilized in this study consisted of an HTC Vive Pro Eye head-mounted display (HTC Corporation, Taoyuan, Taiwan) equipped with a built-in Tobii eye tracker (Tobii Technology, Stockholm, Sweden). The system also included two base stations, two VR controllers, and two additional body trackers affixed to the feet for comprehensive full-body motion tracking.

Software. The VR environment was developed using Unity 3D 5.6.1 software (Unity Technologies, San Francisco, CA, USA) and featured a room with a mirror placed 1.5 virtual meters in front of the patient, along with two boxes placed on the floor. The patients were able to observe their entire body from a first-person perspective and their reflection in the mirror, even while they were in motion. The avatars, created using Blender version 2.78 software, were outfitted with a head-mounted display similar to what the patients wore. They also wore a tank top paired with jeans, allowing for customization of the clothing color to match that of the participants, and black trainers. To minimize the impact of hairstyle, they wore a grey hat covering their hair.

### 2.4. Procedure

This study received approval from the ethics committees of both the University of Barcelona and the Hospital Sant Joan de Déu of Barcelona. Written informed consent was obtained from both the participants and their legal guardians. Personalized avatars were crafted by overlaying frontal and lateral photographs of each patient onto a virtual silhouette. The avatar’s height and body proportions were then adjusted to match those of the respective patient’s silhouette.

During this process, the patient initially completed version A of the BIAS-BD questionnaire. Following questionnaire completion, the patient was then immersed in the virtual environment. Upon entering the room, a five-minute visuo-motor and visuo-tactile stimulation protocol, adapted from previous studies [7,45,46], was applied to induce a full-body ownership illusion (FBOI), increasing participants’ identification with the virtual body [47]. Patients were given instructions to maintain their gaze on their own reflection in the mirror without making any movements for 30 s, during which their eye movements were recorded. To mitigate potential bias arising from knowledge of the true objective, this pre-training AB assessment was presented to the patients as a sensor calibration task. The true purpose of the assessment was only disclosed after the session had concluded.

**Figure 2:**
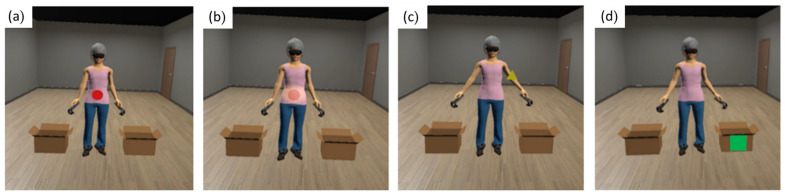
Attentional bias modification training visual representation: geometric figures appearing on a weight-related body part (**a**), on an illuminated weight-related body part (**b**), on a non-weight-related body part (**c**), and on neutral stimulus (**d**).

The next step was the ABMT, which was derived from a modified version of the attention bias induction procedure introduced by Smeets et al. [26] and developed through the visual selection of geometric figures with various colors that fitted specific body areas. Participants were explicitly informed of the real goal of the training (learning to pay attention to all body parts), because this could potentially enhance the learning process by engaging both implicit and explicit aspects of the task [48,49]. Specifically, participants were asked to stare for 4 s at the specific body part where the geometrical figures appeared on the avatar, while it was progressively illuminated until the end of the 4 s, and then to move on to the next figure presentation. To ensure that all patients had spent the same amount of time on all parts of the body by correctly completing the entire task, if the patient deviated their gaze from the stimulus, the system blocked the elapsing of seconds until the participant returned the gaze to the stimulus. To make the ABMT more interactive and to maintain the motivation to perform the task, participants were asked to detect and identify the geometric figures through a figure discrimination task based on naming the figure’s shape in half of the trials and naming the figure’s color in the remaining trials. During the training, the geometric figures were displayed in 45% of the trials on body parts related to weight. In another 45% of the trials, the figures were presented on body parts related to non-weight. The remaining 10% of the trials featured the figures appearing on two neutral stimuli located adjacent to the avatar (Figure 2).

The division of target stimuli into specific percentages in the ABMT serves several purposes. The primary goal of ABMT was to achieve a balanced allocation of attention between weight-related and non-weight-related body parts. To ensure this equilibrium, the stimuli were distributed almost equally, with 45% allocated to each of these two categories. This distribution guarantees that participants receive training in giving equal attention to both types of body parts. The inclusion of 10% neutral stimuli in ABMT extends its effects beyond training, promoting the transfer of balanced attention allocation from a training context to real-world situations by introducing variability. Also, it serves to maintain engagement and focus during the training. When stimuli are heavily biased toward one category, participants might anticipate the location of the next stimulus, influencing training outcomes. By distributing stimuli between weight-related, non-weight-related body parts, and neutral stimuli, ABMT keeps participants engaged and attentive throughout the training.

The patients performed the search-and-stare task for a total of 150 figures, which were split into two blocks of 75 figures each. A one-minute break was provided between the two blocks, resulting in a total task duration of approximately 10 to 15 min. The duration of the task in this study was determined based on the findings from a previous study that examined the optimal task duration for the ABMT in healthy women. The established duration aimed to ensure effectiveness while aligning with the previous research’s findings [36]. Finally, during the post-training assessment, ET measures were taken again with the same cover story as before, and, once the VR headset and trackers were removed, the participant completed version B of the BIAS-BD.

### 2.5. Statistical Analyses

The Open Gaze and Mouse Analyzer (OGAMA; Freie Universität, Berlin, Germany) analysis software was used to convert raw ET data into appropriate quantitative data. Additional data processing involved the calculation of the difference between weight-related and non-weight-related AOIs. For example, in terms of the fixations number, it resulted in 25 (30 fixations to weight-related AOIs—5 fixations to non-weight-related AOIs). Therefore, a score close to 0 indicates balanced attention between weight and non-weight-related body parts, while a positive score indicates greater attention to weight-related body parts, and a negative score indicates greater attention to non-weight-related body parts. The outcomes of the intervention were analyzed by the statistical software IBM SPSS Statistics v.28. The Shapiro–Wilk test did not show evidence of non-normality for both body dissatisfaction and complete fixation time variables. Based on these results, a paired samples *t*-test was used to determine whether there was a statistically significant difference in the BD and complete fixation time measures between pre- and post-treatment assessments. However, the distribution of the number of fixation variables departed significantly from normality at the pre-treatment assessment but not for the post-treatment assessment. Therefore, a Wilcoxon signed-rank test was used to determine whether there was a statistically significant difference in the number of fixations before and after the training.

## 3. Results

The clinical sample consisted of 23 AN female adolescents. Table 1 provides the clinical characteristics of the patients, including the subtype of anorexia nervosa, comorbidities, and the types of pharmacological treatment received.

**Figure 3:**
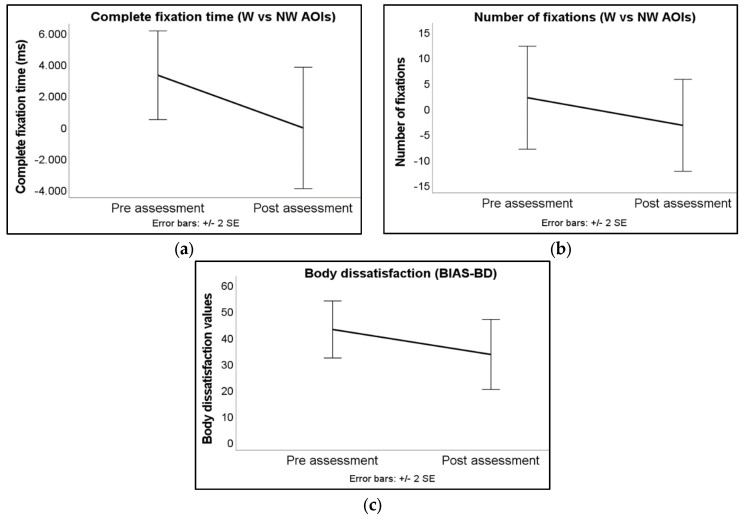
Means of the patients at the two assessment conditions (pre-assessment, post-assessment) in complete fixation time (**a**), number of fixations (**b**), and body dissatisfaction (**c**). Error bars represent 95% confidence intervals (+/− 2 SE). Note: “W vs. NW AOIs” = weight vs. non-weight areas of interest; “BIAS-BD” = Body Image Assessment Scale-Body Dimensions.

Figure 3a–c displays the mean values and 95% confidence intervals for BD and body-related AB measures at both the pre-training assessment and post-training assessment time points.

A one-tailed paired samples *t*-test was performed to analyze whether patients had lower complete fixation time and BD levels after the ABMT (see Table 2).

A Wilcoxon signed-rank test was used to determine whether there was a statistically significant reduction in the number of fixations following the training (see Table 3).

Attentional bias measures. There was a significant reduction in the complete fixation time on the weight-related AOIs at the post-training assessment compared to the pre-training assessment, resulting in a balanced complete fixation time between the weight-related and non-weight-related AOIs. There was no statistically significant change in the number of fixations.

Body dissatisfaction. A significant decrease in BD was observed in the post-training assessment time compared to the pre-training assessment time.

## 4. Discussion

The main goal of this study was to investigate the effectiveness of a single session of ABMT using VR and ET technology in reducing body-related AB and BD in adolescent patients with AN.

This study confirmed that patients with AN had AB towards weight-related body parts, as indicated by a higher fixation time on these areas. This aligns with previous research [7,44,50] suggesting that AB can act as a maintaining factor in disorders through cognitive and emotional mechanisms [8]. Selective processing of symptom-relevant stimuli leads to disregarding conflicting information [51], especially when those stimuli are perceived as threatening, triggering a vicious circle of hypervigilance and exclusion of disconfirming stimuli [52,53]. As expected, the ABMT restored balanced attention between weight- and non-weight-related body areas by reducing the complete fixation time on weight-related body parts. These findings align with previous studies that demonstrated the effectiveness of ABMT in reducing appearance bias [22,24] but contrast with a study that did not elicit AB change [23]. The contrasting results with this other study may be attributed to methodological differences. This study was carried out by combining VR and ET technologies, and the ABMT was more focused on balancing attention between positive (non-weight-related body parts) and negative (weight-related body areas) stimuli using a virtual representation of the patient’s real body parts, while the other study used a computer dot-probe task to induce AB towards a specific stimuli valence using words related to appearance, body shape, and food in a positive, negative, or neutral connotation.

In contrast to the complete fixation time measure, AN patients showed no body-related AB when the fixations number measure was considered a measure of AB, as participants exhibited a balanced number of fixations between weight and non-weight-related body parts at baseline, which remained unchanged after the ABMT, indicating that the ABMT did not impact the fixations number due to the absence of bias to correct.

The measures of the complete fixation time and fixations number were both used to assess the level of attention and cognitive processing in the AOIs, but their interpretation may differ. While the fixations number reflects the semantic importance of stimuli [54,55], complete fixation time is influenced by the complexity and level of interest in the AOI [56,57]. Therefore, participants may distribute their fixations number between weight and non-weight-related body parts because both belong to the semantic category of the body, which is clinically significant for patients with AN. The higher complete fixation time spent on weight-related areas at baseline may be attributed to their emotional relevance and complexity, suggesting a deeper processing of information related to those areas.

The interpretation of the change in the complete fixation time pattern after ABMT must be considered carefully, as these changes may depend on the interplay between automatic attentional processes, higher-order attentional control mechanisms, and goal-directed behavior [58,59,60]. Threat stimuli might be predominantly processed through automatic processes, whereas neutral stimuli may necessitate a certain level of attentional control for sufficient processing [61,62]. ABMT aims to teach attention control [63], and it is possible that patients, knowing the goal of the training, voluntarily chose to allocate attention to non-weight-related body areas in addition to automatic attention to weight-related areas. The development of attention control abilities involves dedicated neural architecture and multiple neural pathways, which are influenced by repeated exposure to specific tasks, such as ABMT [64,65,66]. However, further information is needed to determine whether the balanced complete fixation time after the ABMT reflects learned attention control, changes in emotional and cognitive relevance of non-weight-related body areas, or a combination of these factors.

Furthermore, this study demonstrated a reduction in BD levels reported by patients after ABMT. These findings align with previous research by Smith and Rieger [24], who observed that inducing AB towards negative body-related stimuli increased BD and induced an AB towards the respective target stimuli. Our findings are also consistent with those reported by Smeets et al. [26], who found that inducing an AB for self-defined unattractive body parts led to a reduction in body satisfaction, whereas inducing an AB for self-defined attractive body parts led to an increase in body satisfaction. These findings highlight the role of body-related AB in maintaining body image disturbances [5,9] and suggest that body image-related AB and levels of BD can be manipulated. If AB exacerbates levels of BD, AB could be an appropriate target for interventions aimed at reducing BD. Redirecting attention towards both weight- and non-weight-related body parts could be beneficial for improving BD and developing alternative cognitive and behavioral patterns. In contrast with our study, studies by Loughnan et al. [23] and Allen et al. [22], using neutral and positive appearance-based ABMT, respectively, did not effectively reduce BD nor elicit AB towards specific targets. These results support the hypothesis that changes in AB can influence symptom changes: when bias was successfully modified, symptomatology also changed; conversely, unsuccessfully modifying bias resulted in no symptom change [67,68,69].

Based on previous studies, it is hypothesized that AB plays a significant role in the development and maintenance of BD in individuals with AN and healthy women [5,9,50]. ABMT has important clinical implications, since it holds the potential for the prevention and treatment of eating disorders by modifying AB [6,16]. Additionally, ABMT has the potential to directly influence subcortical attentional processes and cognitive operations that operate beyond conscious control, making it a potentially effective approach for patients with persistent forms of the disease who may not benefit from traditional “top-down” cognitive therapies [16].

Implementing VR equipment in eating disorder services can present challenges due to the costs and logistical considerations involved. However, the use of VR technology in ABMT offers advantages in terms of flexibility and acceptability. The virtual nature of the task allows for the incorporation of diverse threat stimuli, enhancing the customization of interventions. Moreover, the immersive and interactive nature of VR increases patient engagement and motivation, contributing to more effective training outcomes. Despite the challenges, the benefits of using VR in ABMT make it a promising approach to eating disorder treatment [70].

Although the significant efficacy of the ABMT developed and analyzed in this study is noteworthy, several limitations need to be addressed. These include a small sample size and the absence of a control group. Additionally, this study only measured acute effects without assessing long-term outcomes. Participants reported that ABMT was repetitive in nature, suggesting the potential for enhancing user engagement by introducing gamified features such as rewards and animations [71]. Future research should replicate this study with a larger sample size, evaluate the long-term effects of ABMT through follow-up assessments, and investigate whether multiple sessions or combining ABMT with traditional psychological therapy, such as mirror exposure therapy, can improve its effectiveness. Furthermore, it is important to acknowledge that the clinical heterogeneity within our sample could have influenced the results. AN is characterized by a spectrum of clinical presentations, including differences in symptom severity, duration of illness, and the presence of comorbid conditions. Future research should consider stratifying samples based on clinical phenotypes to explore potential differential treatment responses. Finally, it would be valuable to explore the preventive potential of ABMT for women at risk of developing eating disorders and extend the research to include the male population, who are also experiencing an increase in body image disturbances and eating disorders [72,73].

## 5. Conclusions

This study showcases the promising potential of a novel ABMT based on VR and ET as a valuable clinical tool in reducing body-related AB and BD in adolescent patients diagnosed with AN. By simulating the image of the patient reflected in a mirror and utilizing objective measurements of visual attention patterns, this ABMT offers a meaningful and effective approach to address these core challenges in AN treatment. The findings suggest that this intervention can enhance therapeutic outcomes and contribute to the overall well-being of adolescent patients with AN.

## Figures and Tables

**Table 1:** Clinical characteristics of patients.

Clinical Characteristic	Number of Patients
Subtype of anorexia nervosa	
Restrictive Anorexia Nervosa	22
Purgative Anorexia Nervosa	1
Comorbidities	
Major Depressive Disorder	2
Major Depressive Disorder and Mild Intellectual Disability	1
Post-Traumatic Stress Disorder	1
Social Anxiety Disorder	2
Obsessive Compulsive Disorder	1
Pharmacological Treatment	
Antipsychotics	2
Antidepressants	11
Anxiolytics	2
Antipsychotics and Antidepressants	8
Antipsychotics and Anxiolytics	1
Antidepressants and Anxiolytics	4
Antidepressants, Anxiolytics, and Antipsychotics	4

**Table 2:** Paired samples *t*-test comparing complete fixation time and body dissatisfaction between pre-assessment and post-assessment time.

	Pre-Assessment Time	Post-Assessment Time	Paired Samples *t*-Test		Effect Size
	Mean (SD)	Mean (SD)	*t*	*p*	Cohen’s d *
Complete fixation time (in ms)	3269.88 (5837.05)	−94.88 (7988.81)	1.863	* 0.040	0.452
Body dissatisfaction	42.83 (26.14)	33.26 (32.14)	1.880	* 0.037	0.392

Note: Significant differences. * *p* < 0.05; Cohen’s d effect sizes: small (≥0.20), medium (≥0.50), and large (≥0.80).

**Table 3:** Wilcoxon signed-rank test comparing the number of fixations between pre-assessment and post-assessment time.

	Pre-Assessment Time	Post-Assessment Time	Wilcoxon Signed-Rank Test		Effect Size
	Mean (SD)	Mean (SD)	*z*	*p*	*r*^2^ *
Number of fixations	2.00 (20.80)	−3.41 (18.56)	−0.592	0.554	−0.100

Note: * R-squared effect size: small (≥0.10), medium (≥0.30) and large (≥0.50).

## Data Availability

The data presented in this study are available on request from the corresponding author.

## References

[1] Crow S. (2013). Eating disorders and risk of death. Am. J. Psychiatry.

[2] Favaro A., Caregaro L., Tenconi E., Bosello R., Santonastaso P. (2009). Time trends in age at onset of anorexia nervosa and bulimia nervosa. J. Clin. Psychiatry.

[3] Goschke T. (2014). Dysfunctions of decision-making and cognitive control as transdiagnostic mechanisms of mental disorders: Advances, gaps, and needs in current research. Int. J. Methods Psychiatr. Res..

[4] Lee M., Shafran R. (2004). Information processing biases in eating disorders. Clin. Psychol. Rev..

[5] Williamson D.A., White M.A., York-Crowe E., Stewart T.M. (2004). Cognitive-behavioral theories of eating disorders. Behav. Modif..

[6] Bar-Haim Y., Lamy D., Pergamin L., Bakermans-Kranenburg M.J., van IJzendoorn M.H. (2007). Threat-related attentional bias in anxious and nonanxious individuals: A meta-analytic study. Psychol. Bull..

[7] Porras-Garcia B., Ferrer-Garcia M., Serrano-Troncoso E., Carulla-Roig M., Soto-Usera P., Miquel-Nabau H., Olivares L., Marnet-Fiol R., Santos-Carrasco I.M., Borszewski B. (2021). AN-VR-BE. A randomized controlled trial for reducing fear of gaining weight and other eating disorder symptoms in anorexia nervosa through virtual reality-based body exposure. J. Clin. Med..

[8] Battagliese G., Lombardo C. (2011). Attentional bias in psychopathology. Psicoter. Cogn. E Comport..

[9] Rodgers R.F., DuBois R.H. (2016). Cognitive biases to appearance-related stimuli in body dissatisfaction: A systematic review. Clin. Psychol. Rev..

[10] Stice E., Ng J., Shaw H. (2010). Risk factors and prodromal eating pathology. J. Child Psychol. Psychiatry.

[11] Smith E., Rieger E. (2006). The effect of attentional bias toward shape- and weight-related information on body dissatisfaction. Int. J. Eat. Disord..

[12] Glashouwer K.A., Masselman I., de Jong P.J. (2019). Reducing body dissatisfaction by means of an evaluative conditioning procedure in undergraduate women: A replication study. Behav. Res. Ther..

[13] Cass J., Giltrap G., Talbot D. (2020). Female body dissatisfaction and attentional bias to body images evaluated using visual search. Front. Psychol..

[14] Griffen T.C., Naumann E., Hildebrandt T. (2018). Mirror exposure therapy for body image disturbances and eating disorders: A review. Clin. Psychol. Rev..

[15] Ferrer-Garcia M., Porras-Garcia B., Miquel H., Serrano-Troncoso E., Carulla-Roig M., Gutiérrez J. (2021). The way we look at our own body really matters! Body-related attentional bias as a predictor of worse clinical outcomes after a virtual reality body exposure therapy. Annu. Rev. Cybertherapy Telemed..

[16] Renwick B., Campbell I.C., Schmidt U. (2013). Attention bias modification: A new approach to the treatment of eating disorders?. Int. J. Eat. Disord..

[17] Bar-Haim Y. (2010). Research review: Attention bias modification (ABM): A novel treatment for anxiety disorders. J. Child Psychol. Psychiatry.

[18] Hallion L.S., Ruscio A.M. (2011). A meta-analysis of the effect of cognitive bias modification on anxiety and depression. Psychol. Bull..

[19] Dikstein H., Gilon-Mann T., Halevi-Yosef R., Enoch-Levi A., Hamdan S., Gur E., Haim Y.B., Lazarov A., Treasure J., Stein D. (2023). Attention bias modification add-on to inpatient treatment for young women with anorexia nervosa—A randomized controlled trial. Eur. Eat. Disord. Rev..

[20] Brockmeyer T., Friederich H.C., Küppers C., Chowdhury S., Harms L., Simmonds J., Gordon G., Potterton R., Schmidt U. (2019). Approach bias modification training in bulimia nervosa and binge-eating disorder: A pilot randomized controlled trial. Int. J. Eat. Disord..

[21] Schmitz F., Svaldi J. (2017). Effects of bias modification training in binge eating disorder. Behav. Ther..

[22] Allen L., Mulgrew K.E., Rune K., Allen A. (2018). Attention bias for appearance words can be reduced in women: Results from a single-session attention bias modification task. J. Behav. Ther. Exp. Psychiatry.

[23] Loughnan S.A., Mulgrew K.E., Lane B.R. (2015). Attention bias modification produces no changes to appearance-related bias, state or trait body dissatisfaction in nonclinical women. Health Psychol. Open.

[24] Smith E., Rieger E. (2009). The effect of attentional training on body dissatisfaction and dietary restriction. Eur. Eat. Disord. Rev..

[25] Engel S.G., Robinson M.D., Wonderlich S.J., Meier B.P., Wonderlich S.A., Crosby R.D., Steffen K.J., Mitchell J.E. (2006). Does the avoidance of body and shape concerns reinforce eating disordered attitudes? Evidence from a manipulation study. Eat. Behav..

[26] Smeets E., Jansen A., Roefs A. (2011). Bias for the (un)attractive self: On the role of attention in causing body (dis)satisfaction. Health Psychol..

[27] MacLeod C., Mathews A., Tata P. (1986). Attentional bias in emotional disorders. J. Abnorm. Psychol..

[28] Hertel P.T., Mathews A. (2011). Cognitive bias modification: Past perspectives, current findings, and future applications. Perspect. Psychol. Sci..

[29] Heeren A., Mogoașe C., Philippot P., McNally R.J. (2015). Attention bias modification for social anxiety: A systematic review and meta-analysis. Clin. Psychol. Rev..

[30] Clus D., Larsen M.E., Lemey C., Berrouiguet S. (2018). The use of virtual reality in patients with eating disorders: Systematic review. J. Med. Internet Res..

[31] Perpiñá C., Botella C., Baños R.M. (2003). Virtual reality in eating disorders. Eur. Eat. Disord. Rev..

[32] Gutiérrez-Maldonado J., Ferrer-García M., Dakanalis A., Riva G., Agras W.S., Robinson A.H. (2018). Virtual reality: Applications to eating disorders. The Oxford Handbook of Eating Disorders.

[33] Pla-Sanjuanelo J., Ferrer-García M., Vilalta-Abella F., Riva G., Dakanalis A., Ribas-Sabaté J., Andreu-Gracia A., Fernandez-Aranda F., Sanchez-Diaz I., Escandón-Nagel N. (2017). Testing virtual reality-based cue-exposure software: Which cue-elicited responses best discriminate between patients with eating disorders and healthy controls?. Eat. Weight. Disord..

[34] Kerr-Gaffney J., Harrison A., Tchanturia K. (2018). Eye-tracking research in eating disorders: A systematic review. Int. J. Eat. Disord..

[35] Miquel-Nabau H., Briseño-Oloriz N., Porras-Garcia B., Ascione M., Meschberger-Annweiler F.A., Ferrer-Garcia M., Moreno-Sanchez M., Serrano-Troncoso E., Carulla-Roig M., Gutiérrez Maldonado J. (2023). Modification of body-related attentional bias through virtual reality and eye-tracking in healthy participants: Implications for anorexia nervosa treatments. Brain Sci..

[36] Meschberger-Annweiler F.A., Ascione M., Porras-Garcia B., Ferrer-Garcia M., Moreno-Sanchez M., Miquel-Nabau H., Serrano-Troncoso E., Carulla-Roig M., Gutiérrez-Maldonado J. (2023). An attentional bias modification task, through virtual reality and eye-tracking technologies, to enhance the treatment of anorexia nervosa. J. Clin. Med..

[37] Herpertz-Dahlmann B., Dempfle A., Egberts K.M., Kappel V., Konrad K., Vloet J.A., Bühren K. (2018). Outcome of childhood anorexia nervosa—The results of a five- to ten-year follow-up study. Int. J. Eat. Disord..

[38] American Psychiatric Association (2013). Diagnostic and Statistical Manual of Mental Disorders.

[39] World Health Organization (2006). WHO Child Growth Standards: Length/Height-Forage, Weight-for-Age, Weight-for-Length, Weight-for-Height and Body Mass Index-for-Age: Methods and Development.

[40] Gardner R.M., Jappe L.M., Gardner L. (2009). Development and validation of a new figural drawing scale for body-image assessment: The BIAS-BD. J. Clin. Psychol..

[41] Reed D.L., Thompson J.K., Brannick M.T., Sacco W.P. (1991). Development and validation of the Physical Appearance State and Trait Anxiety Scale (PASTAS). J. Anxiety Disord..

[42] Jacob R.J.K., Karn K.S., Hyönä J., Radach R., Deubel H. (2003). Eye tracking in human-computer interaction and usability research: Ready to deliver the promises. The Mind’s Eye: Cognitive and Applied Aspects of Eye Movement Research.

[43] Jiang M.Y.W., Vartanian L.R. (2018). A review of existing measures of attentional biases in body image and eating disorders research: Attentional measures in body image research. Aust. J. Psychol..

[44] Bauer A., Schneider S., Waldorf M., Braks K., Huber T.J., Adolph D., Vocks S. (2017). Selective visual attention towards oneself and associated state body satisfaction: An eye-tracking study in adolescents with different types of eating disorders. J. Abnorm. Child Psychol..

[45] Porras-Garcia B., Ferrer-Garcia M., Yilmaz L., Sen Y.O., Olszewska A., Ghita A., Serrano-Troncoso E., Treasure J., Gutiérrez-Maldonado J. (2020). Body-related attentional bias as mediator of the relationship between body mass index and body dissatisfaction. Eur. Eat. Disord. Rev..

[46] Waltemate T., Gall D., Roth D., Botsch M., Latoschik M.E. (2018). The impact of avatar personalization and immersion on virtual body ownership, presence, and emotional response. IEEE Trans. Vis. Comput. Graph..

[47] Wiederhold B.K., Riva G., Gutiérrez-Maldonado J. (2016). Virtual reality in the assessment and treatment of weight-related disorders. Cyberpsychol. Behav. Soc. Netw..

[48] Lazarov A., Abend R., Seidner S., Pine D.S., Bar-Haim Y. (2017). The effects of training contingency awareness during attention bias modification on learning and stress reactivity. Behav. Ther..

[49] MacLeod C., Koster E.H., Fox E. (2009). Whither cognitive bias modification research? Commentary on the special section articles. J. Abnorm. Psychol..

[50] Tuschen-Caffier B., Bender C., Caffier D., Klenner K., Braks K., Svaldi J. (2015). Selective visual attention during mirror exposure in anorexia and bulimia nervosa. PLoS ONE.

[51] Beck A.T. (1976). Cognitive Therapy and the Emotional Disorders.

[52] Clark D.M. (1999). Anxiety disorders: Why they persist and how to treat them. Behav. Res. Ther..

[53] Mogg K., Bradley B.P. (1998). A cognitive-motivational analysis of anxiety. Behav. Res. Ther..

[54] Poole A., Ball L.J., Phillips P., Fincher S., Markopolous P., Moore D., Ruddle R. (2004). In search of salience: A response time and eye movement analysis of bookmark recognition. People and Computers XVIII-Design for Life: Proceedings of HCI 2004.

[55] Yarbus A.L. (1967). Eye Movements and Vision.

[56] Fisher D.F., Monty R.A., Senders J.W. (2017). Eye Movements: Cognition and Visual Perception.

[57] Goldberg J.H., Kotval X.P. (1999). Computer interface evaluation using eye movements: Methods and constructs. Int. J. Ind. Ergon..

[58] LeDoux J.E., Pine D.S. (2016). Using neuroscience to help understand fear and anxiety: A two-system framework. Am. J. Psychiatry.

[59] Shechner T., Bar-Haim Y. (2016). Threat monitoring and attention-bias modification in anxiety and stress-related disorders. Curr. Dir. Psychol. Sci..

[60] Corbetta M., Shulman G.L. (2002). Control of goal-directed and stimulus-driven attention in the brain. Nat. Rev. Neurosci..

[61] Yiend J. (2010). The effects of emotion on attention: A review of attentional processing of emotional information. Cogn. Emot..

[62] Eysenck M.W., Derakshan N., Santos R., Calvo M.G. (2007). Anxiety and cognitive performance: Attentional control theory. Emotion.

[63] Hakamata Y., Lissek S., Bar-Haim Y., Britton J.C., Fox N.A., Leibenluft E., Ernst M., Pine D.S. (2010). Attention bias modification treatment: A meta-analysis toward the establishment of novel treatment for anxiety. Biol. Psychiatry.

[64] Balleine B.W., O’Doherty J.P. (2010). Human and rodent homologies in action control: Corticostriatal determinants of goal-directed and habitual action. Neuropsychopharmacology.

[65] Pine D.S., Helfinstein S.M., Bar-Haim Y., Nelson E., Fox N.A. (2009). Challenges in developing novel treatments for childhood disorders: Lessons from research on anxiety. Neuropsychopharmacology.

[66] Browning M., Holmes E.A., Murphy S.E., Goodwin G.M., Harmer C.J. (2009). Lateral prefrontal cortex mediates the cognitive modification of attentional bias. Biol. Psychiatry.

[67] Grafton B., MacLeod C., Rudaizky D., Holmes E.A., Salemink E., Fox E., Notebaert L. (2017). Confusing procedures with process when appraising the impact of cognitive bias modification on emotional vulnerability. Br. J. Psychiatry.

[68] Kuckertz J.M., Amir N. (2015). Attention bias modification for anxiety and phobias: Current status and future directions. Curr. Psychiatry Rep..

[69] MacLeod C., Clarke P.J.F. (2015). The attentional bias modification approach to anxiety intervention. Clin. Psychol. Sci..

[70] Mouatt B., Smith A., Mellow M., Parfitt G., Smith R., Stanton T. (2020). The use of virtual reality to influence motivation, affect, enjoyment, and engagement during exercise: A scoping review. Front. Virtual Real..

[71] Boendermaker W.J., Prins P.J., Wiers R.W. (2015). Cognitive bias modification for adolescents with substance use problems—Can serious games help?. J. Behav. Ther. Exp. Psychiatry.

[72] Gorrell S., Murray S.B. (2019). Eating disorders in males. Child Adolesc. Psychiatr. Clin..

[73] Fiske L., Fallon E.A., Blissmer B., Redding C.A. (2014). Prevalence of body dissatisfaction among United States adults: Review and recommendations for future research. Eat. Behav..

